# Investigating Relationships between Preschool Children’s Perceived Competence, Motor Skills, and Physical Activity: A Cross-Lagged Panel Model

**DOI:** 10.3390/jcm10235620

**Published:** 2021-11-29

**Authors:** Suryeon Ryu, Jung Eun Lee, Wenxi Liu, Daniel J. McDonough, Zan Gao

**Affiliations:** 1 School of Kinesiology, The University of Minnesota–Twin Cities, 1900 University Ave. SE, Minneapolis, MN 55455, USA; ryu00025@umn.edu; 2 Department of Applied Human Sciences, University of Minnesota–Duluth, 1216 Ordean Court SpHC 109, Duluth, MN 55812, USA; junelee@d.umn.edu; 3 Department of Physical Education, Shanghai Jiao Tong University, Shanghai 200240, China; liux4443@umn.edu; 4 School of Public Health, The University of Minnesota–Twin Cities, 420 Delaware St. SE, Minneapolis, MN 55455, USA; mcdo0785@umn.edu

**Keywords:** childhood obesity, locomotor skills, moderate-to-vigorous physical activity, motor skill competence, object control skills

## Abstract

Background: Motor skill competence (MSC) and perceived competence (PC) are primary correlates that are linked with physical activity (PA) participation, yet there is limited evidence of the mutual longitudinal or temporal associations between these variables in preschoolers. Therefore, this study’s purpose was to examine the bidirectional relationships between MSC and PA, MSC and PC, and PC and PA in preschoolers over time. Methods: The final sample were 61 preschoolers (M_age_ = 4.45 years, ranging from 4 to 5) from two underserved schools. MSC was assessed using the Test of Gross Motor Development, Second Edition (TGMD-2). PC was assessed using the Pictorial Scale of Perceived Competence and Social Acceptance for Young Children. PA was assessed using ActiGraph GT9X Link accelerometers during three consecutive school days. All assessments of MSC, PC, and PA were measured in identical conditions at schools at the baseline (T1) and the end of the eighth week (T2). We employed a cross-lagged model approach to understand the bidirectional relationships between MSC, PC, and PA. Results: The results showed that T1 MSC significantly predicted T2 MSC (*p* < 0.01) and T1 MSC significantly predicted T2 PA only in girls (*p* = 0.03). Additionally, a cross-lagged effect of T1 MSC and T2 PC was only observed in boys (*p* = 0.03). Lastly, a significant association for T1 moderate-to-vigorous physical activity (MVPA) and T2 PC was only observed in girls (*p* = 0.04). Conclusions: Bidirectional relationships between the variables were not observed in preschoolers. However, significant gender differences were observed in each cross-lagged model.

## 1. Introduction

Physical activity (PA) participation plays a crucial role in preventing obesity and promoting cardiovascular fitness among young children (i.e., those 4–6 years of age) [1]. Alarmingly, the global prevalence of childhood overweight and obesity has increased from only 4% in 1975 to 18% in 2016, indicating that over 124 million children and adolescents aged 5–19 had overweight or obesity [2]. This is concerning given that early childhood obesity is a significant predictor of adolescent and adult obesity [3,4,5,6], which is associated with increased risk of chronic diseases, such as heart disease and type 2 diabetes [2]. That is, early childhood (aged between 4 and 6 years) has been recognized as a critical period to promote and develop healthy PA behaviors that track through the lifespan [3,4,5,6], which may lead to long-term health benefits [1].

As one of the important correlates of PA [7], motor skill competence (MSC) refers to proficiency in object control skills (e.g., catching, throwing, kicking) and locomotor skills development (e.g., running, hopping, sliding), which are a group of fundamental motor skills that are necessary to acquire to successfully participate in sports or physical activity [7,8]. Research has shown that motor skills acquisition in early childhood is an important prerequisite for children to participate in PA and maintain physically active lifestyles [9,10,11]. Given that young children’s MSC tends to improve as they age [12] and that learning to move is a necessary skill underlying future PA [13], more advanced MSC was hypothesized to be a crucial determinant of healthy PA behaviors among young children [7,14].

In addition to MSC, perceived competence (PC) is also associated with PA participation in young children [15,16,17,18,19]. Indeed, Harter [19], using competent motivation theory, found that PC and mastery competence are predictors that can explain children’s PA participation [1]. PC is defined in terms of judgments that children possess about their skills to achieve specific movement tasks [17,20]. Indeed, during PA participation, children with greater PC demonstrate greater patience and make attempts to be proficient at different PA skills or tasks compared with those who perceive themselves to be less physically competent, which results in a loss of interest and, therefore, does not foster favorable long-term PA behaviors [21,22]. In addition, children’s PC is suggested to positively predict their intrinsic motivation [23], which leads children to be physically active and less anxious during PA participation [20,21].

According to the Stodden et al. [7] conceptual model, MSC and PC are the primary mechanisms that are linked to PA participation and health-related physical fitness. In fact, robust research evidence has shown that PC, along with fundamental MSC, could contribute to the health of young children given their potential effects on PA participation [22,24] and health-related physical fitness [25]. Although MSC and PC offer insight into PA promotion, most empirical studies to date have targeted older children and adolescents [1], thereby missing the opportunity to promote these competencies in early childhood, which is a critical period for establishing and developing healthy PA behaviors.

Furthermore, empirical studies reported gender differences in children’s PA, MSC, and PC. In general, empirical evidence suggested that boys exhibited higher PA levels, MSC, and PC than girls [24,25,26,27,28,29]. In detail, boys had higher PA levels than girls [26,27]. MSC was also found to be greater among boys compared with girls. Boys were especially more proficient in object control motor skills [22,28,29] and locomotor skills [22] than girls and also demonstrated greater PC than girls [30,31]. Nevertheless, the moderation effect of gender on the relationships between PA, MSC, and PC is not available. In response, this study investigated the gender moderation on the relationships between PA and MSC, MSC and PC, and PA and PC.

Despite previous studies suggesting meaningful relationships between MSC, PC, and PA, investigation of the bidirectional relationships between these variables to evaluate potential mechanisms remains scarce. Notably, although the preschool years (ages 4–6 years) were identified as a crucial period to promote healthy lifestyle habits, few studies have paid attention to the bidirectional relationships between preschool children’s MSC, PC, and PA.

Therefore, the purpose of this study was to investigate the direction of the associations between MSC, PC, and PA in preschool children by using a cross-lagged panel analysis approach, which estimates the structural relations of repeated measure variables [32]. The cross-lagged panel model is a function of the past, which includes autoregressive effects and cross-lagged effects as an essential estimation of the analysis [33]. Autoregressive effects depend on a lagged path from the past variable to the future variable (i.e., past MSC to future MSC). Cross-lagged effects rely on a crossed path from the past of one variable to the future of another variable (i.e., past MSC to future PA) [33]. Based on the findings of past literature on MSC, PC, and PA in children, our first hypothesis was that there would be a positive bidirectional relationship between MSC and moderate-to-vigorous physical activity (MVPA). Next, we hypothesized that there would be a positive bidirectional relationship between MSC and PC. Additionally, it was hypothesized that there would be a positive bidirectional relationship between PC and MVPA. Lastly, there would be gender moderation on the aforementioned relationships. Understanding these potential mutual associations could provide insightful information for developing interventions that are aimed to promote healthy PA behaviors in young children, which may ultimately help to prevent chronic diseases in adulthood. Furthermore, this study is also significant because it could contribute to developing cost-effective PA interventions in cost-conscious health environments, such as schools.

## 2. Methods

### 2.1. Participants and Research Setting

The study was based on secondary data analysis from a parent trial that was conducted in a Mid-Western U.S. state [1]. The participants were a sample of 65 preschool children (33 girls; M_age_ = 4.45 years, ranging from 4 to 5) that were recruited from two underserved urban elementary schools using convenience sampling. Both schools were Title I schools with over 50% children from low-income families. The ethnicity breakdown was as follows: 23 (41.07%) White, 17 (30.36%) Black, 9 (16.07%) Hispanic, 5 (8.93%) Asian, and 2 (3.57%) other. Both schools served preschool to fifth grade with student populations ranging from 500 to 600 and had similar curricula, quality of teachers, and sociocultural environments. Participant inclusion criteria were: (1) children aged 4–5 years, (2) children without diagnosed physical and/or mental disabilities according to school records, and (3) the provision of parental consent forms. The inclusion criteria were verified through the school records and the demographic information sheet. This study was approved by the University of Minnesota Institutional Review Board and school district according to the 1964 Helsinki Declaration and its later amendments or comparable ethical standards [34]. We also obtained parental consent prior to this study. Given that we worked with young children, the University Institutional Review Board exempted the requirement for child assent.

### 2.2. Measures

#### 2.2.1. Demographic and Anthropometric Measurements

The participants’ demographic information, including date of birth, gender, race/ethnicity, and disability status, were collected from school records with the parents’ consent for descriptive purposes. Additionally, height was collected to the nearest half-centimeter using a Seca stadiometer (Seca, Hamburg, Germany) and weight was measured to the nearest 0.1 kg (100 gr) using a Tanita BC-558 IRONMAN^®^ Segmental Body Composition Monitor (Tanita, Tokyo, Japan) digital weight scale. Body mass index (BMI) was calculated using the weight in kilograms divided by the height in meters squared of the children. BMI z-scores were calculated using the built-in function (i.e., zanthro) from STATA (version 15.0; StataCorp, College Station, TX, USA).

#### 2.2.2. Physical Activity Levels

PA was measured using ActiGraph GT9X Link accelerometers (ActiGraph Corps., Pensacola, FL, USA). The ActiGraph GT9X Link is a lightweight, wrist-worn device that resembles a watch and was evident to be a valid and reliable measure of PA in preschool children in school and free-living settings [35,36]. Accelerometers were worn on each participant’s non-dominant wrist during three consecutive school days. Prior to the data collection, each child was assigned an identification number that corresponded with the number on their accelerometer to appropriately keep track of each child’s activity data. Counts were interpreted using empirically based cut-off points that defined the different PA intensities among the preschool children (i.e., sedentary: 0–799 counts per minute, light PA: 800–1679 counts per minute, moderate to-vigorous PA: ≥1680 counts per minute) [37]. All PA data were imported into ActiLife software (ActiGraph Corps., Pensacola, FL, USA; version 6.13) to facilitate the analyses and children’s mean percentage of time spent in moderate-to-vigorous physical activity (MVPA) was the primary outcome variable.

#### 2.2.3. Motor Skill Competence

The Test of Gross Motor Development-2 (TGMD-2) [38], which was demonstrated to be a useful motor skill assessment instrument [39,40], was used to assess MSC. The TGMD-2 is a norm-referenced measure to assess the gross motor skills of children aged 3–10 years, which included 12 skills (six for each subtest): (1) locomotor: running, galloping, hopping, leaping, horizontal jumping, sliding; and (2) object control: striking a stationary ball, stationary dribbling, kicking, catching, overhand throwing, and underhand rolling. The five skills that were examined in this study were selected from these 12 skills, where there were three locomotion skills (running, hopping, jumping) and two objective control skills (kicking and throwing). These skills were included in this study because preschool children were able to perform these skills and it was feasible to test with this age group. In this study, children executed each skill twice. To evaluate the skill performance, qualitative performance criteria were scored, with 1 indicating the presence of a performance criterion for a given motor skill and 0 indicating the absence of the performance criterion. If a skill was assessed using three performance criteria, the raw scores could therefore vary between 0–6. The highest raw total score for the locomotor and the object control skills was 20.

#### 2.2.4. Perceived Competence

The Pictorial Scale of Perceived Competence and Social Acceptance for Young Children [41] was used to assess preschool children’s PC. The pictorial instrument is a valid and reliable measure to assess perceived competence in young children [42]. In detail, the survey has four domains: cognitive competence, physical competence, peer acceptance, and maternal acceptance [41]. Then, children responded to a 5-item perceived physical competence survey using a 4-point Likert-type scale (1 = not too good, 4 = really good). The average score was calculated and used as a measure of each child’s PC. The assessment was individually administered within a private room at each school to protect the children’s privacy.

### 2.3. Procedures

After obtaining parental consent, all assessments were collected based on school schedules and available school spaces. Notably, two trained research assistants administered all assessments. Children’s time spent in MVPA, MSC, and PC were measured within one week at the baseline (T1) and the 8-week follow-up (T2) in 2017–2018. At both time points, all data collection occurred in the school setting. To capture the children’s PA levels, the accelerometers were placed on children’s non-dominant wrists for the entire school day for three consecutive school days. Specifically, each child placed an ActiGraph Link on their wrist when they arrived at school and the accelerometers were collected before classes were finished. To protect privacy, the height, weight, and PC assessments were conducted in private rooms with a single study investigator. MSC testing took approximately 3–5 min per child and was conducted in the school’s gymnasium. If a child was absent when the measurements were being conducted, the data were collected on another day.

### 2.4. Data Analysis

The main variables in the analyses included the T1 and T2 MVPA, T1 and T2 MSC, and T1 and T2 PC. This study employed a cross-lagged model panel approach to discover the dynamic bidirectional relationships between the variables at the two time points (i.e., at the baseline and the 8-week follow-up) [43]. Prior to our primary analyses, outliers were screened using boxplots and a paired t-test was used to compare the variable scores that were retained in the study across the two time points of assessment. Therefore, out of the 65 participants, four children were excluded and a total sample of 61 preschool children was used in the data analysis. Following that, all data analyses were conducted in STATA statistical software (version 15.0; StataCorp, College Station, TX, USA) and the primary analyses were conducted using STATA’s structural equation modeling (SEM) builder. We set the significance level to 0.05 for all main analyses and used effect sizes to compare the mean differences between the two time points, as well as between girls and boys. We reported the effect size as Cohen’s d with small, medium, and large effect sizes defined as d ≤ 0.2, 0.2 < d ≤ 0.5, and 0.5 < d ≤ 0.8, respectively [44,45].

From the complete available sample data, we used three cross-lagged panel models using maximum likelihood to identify the possible bidirectional relationships of the variables over time (i.e., at the baseline and the 8-week follow-up). First, a cross-lagged panel model was implemented to test the relationships between MSC and MVPA at the two time points (T1 and T2). The second model was used to test the relationships between MSC and PC. The third model was used to examine the relationships between PC and MVPA. In addition to the whole-sample analyses, gender stratified analyses were run for all three structural equation models. We report the standardized regression coefficients (β-coefficients) with corresponding 95% confidence intervals (CIs) and the coefficients of determination (R^2^) for all outcomes. Furthermore, we observed the overall model fit of data by using the comparative fit index (CFI) and root-mean-square error of approximation (RMSEA). An RMSEA was classified as excellent if RMSEA < 0.05, good if RMSEA < 0.08, and normal if RMSEA < 0.1; for CFI, it was suggested to be excellent if CFI ≥ 0.90 [43,46,47].

## 3. Results

Descriptive statistics for all variables at both time points by gender are shown in Table 1. We observed no significant mean differences between the two time points (T1 and T2) between all variables, except for MSC (t = −4.45; *p* ≤ 0.00; d = −0.58, large effect). When comparing the mean differences by gender, we observed significant mean differences in the T2 BMI scores and T2 MVPA, with boys having greater BMI scores (t = −2.43, *p* = 0.02, d = −0.62) and spending more time in MVPA compared to girls (t = −3.1818, *p* = 0.023, d = −0.81).

**Figure 1:**
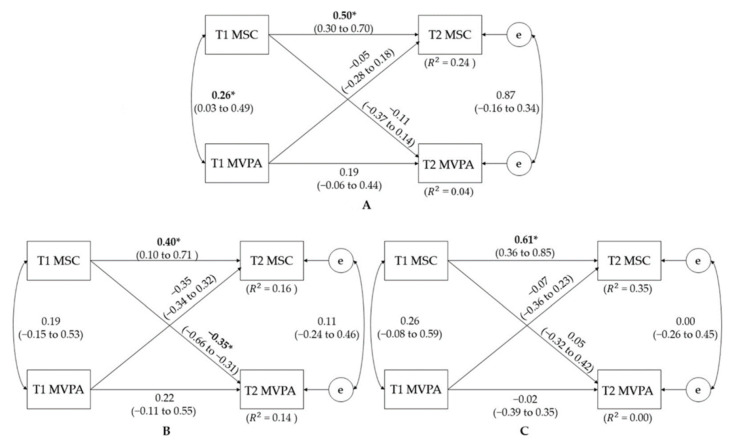
Cross-lagged model of the bidirectional relationships between MSC and total MVPA time before and after the 8-week study period for (**A**) total, (**B**) girls, and (**C**) boys. T1—baseline time; T2—follow-up time; e—error; R^2^ = coefficients of determination; MSC—motor skill competence; MVPA—moderate-to-vigorous physical activity. Path coefficients are standardized with 95% confidence intervals. * denotes a statistically significant path coefficient with *p* < 0.05.

Figure 1 shows the results of the cross-lagged models between MSC and MVPA. Overall, the data fit the model well (CFI = 1.00, RMSEA = 0.00) and the model explained approximately 16 to 35% (R^2^ = 0.16–0.35) and 0 to 14% (R^2^ = 0.00–0.14) of the variances of T2 MSC and MVPA scores when using the baseline (T1) MSC and MVPA scores as predictor variables. When examining the whole sample, T1 MSC significantly correlated with T1 MVPA (*p* = 0.03); however, T2 MSC and T2 MVPA did not indicate a significant correlation. The only significant autoregressive relationship was between T1 MSC and T2 MSC (*p* ≤ 0.01). The cross-lagged effect from T1 MSC to T2 MVPA (*p* = 0.38) and from T1 MVPA to T2 MSC (*p* = 0.66) were not statistically significant. In the gender-specific analyses, most of the relationships were similar to the results from the total sample. However, the significant and negative cross-lagged effect from T1 MSC to T2 MVPA was observed in girls (*p* = 0.03). 

**Figure 2:**
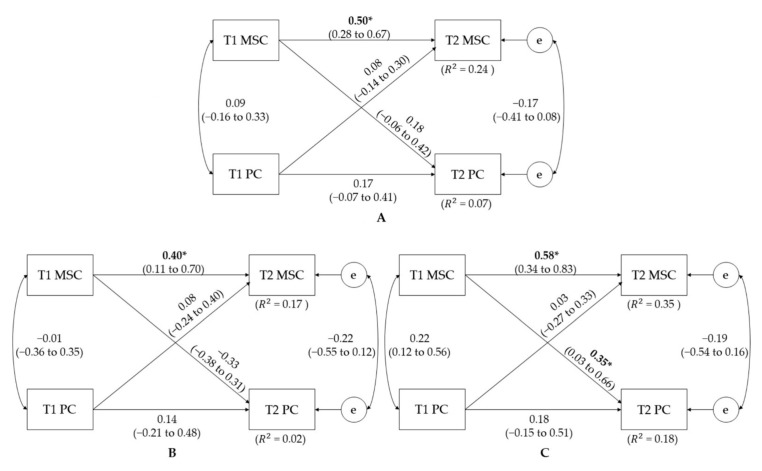
Cross-lagged model of the bidirectional relationships between MSC and PC before and after the 8-week study period for (**A**) total, (**B**) girls, and (**C**) boys. T1—baseline time; T2—follow-up time; PC—perceived competence. Path coefficients are standardized with 95% confidence intervals. * denotes a statistically significant path coefficient with *p* < 0.05.

**Figure 3:**
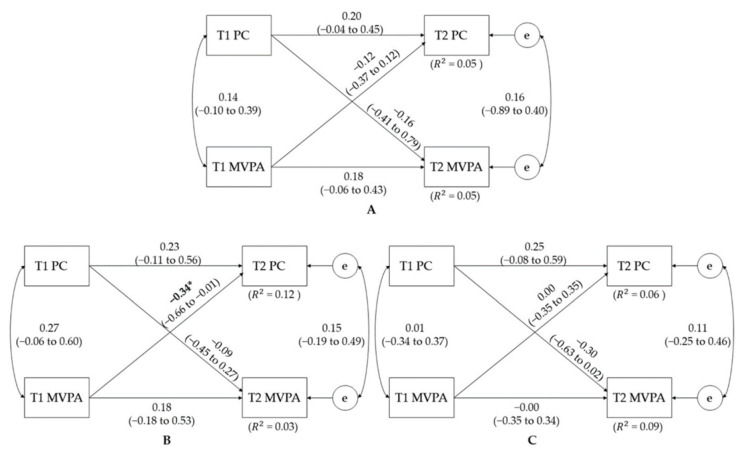
Cross-lagged model of the bidirectional relationships between PC and total MVPA time before and after the 8-week study period for (**A**) total, (**B**) girls, and (**C**) boys. T1—baseline time; T2—follow-up time; MVPA—moderate-to-vigorous physical activity. Path coefficients are standardized with 95% confidence intervals. * denotes a statistically significant path coefficient with *p* < 0.05.

Figure 2 shows the overall cross-lagged models between MSC and PC. The model fit the data well (CFI = 1.00, RMSEA = 0.00) and explained approximately 24 to 35% (R^2^ = 0.24–0.35) and 2 to 18% (R^2^ = 0.02–0.18) of the variances of the T2 MSC and PC scores when using T1 MSC and PC scores as predictor variables. Overall, the significant associations were only detected in the autoregressive pathways. That is, T1 MSC significantly predicted T2 MSC (*p* ≤ 0.01 overall, 0.01 for girls). However, the cross-lagged model for the boys demonstrated a statistically significant cross-lagged pathway from T1 MSC to T2 PC (*p* = 0.03). Figure 3 shows the final cross-lagged models between PC and MVPA. In general, the data fit the model well (CFI = 1.00, RMSEA = 0.00). The model explained approximately 5 to 12% (R^2^ = 0.05–0.12) and 3 to 9% (R^2^ = 0.03–0.09) of the variances of the follow-up (T2) PC and MVPA scores using baseline (T1) PC and MVPA scores as predictor variables. None of the overall autoregressive and cross-lagged relationships were statistically significant, except for the inverse relationship between T1 MVPA and T2 PC among girls (*p* = 0.04).

## 4. Discussion

Despite the well-established importance of PA in preventing and decreasing obesity prevalence in young children, there are limited studies available that identify the correlates and determinants of PA in preschool children. In response, this study investigated the dynamic relationships between MSC and PA, MSC and PC, and PC and PA over 8 weeks to understand the direction and magnitude of the associations between MSC, PC, and PA. We observed gender differences in cross-lagged effects among MSC, PC, and PA, yet none of the bidirectional relationships was reported in all cross-lagged models. Specifically, the gender stratified models between MSC and MVPA suggested significant and reducing prediction from MSC to MVPA in only girls. Further, the gender-specific models between T1 MSC and T2 PC indicated that only boys had significant increasing prediction from T1 MSC to PC. Lastly, the gender stratified models between PC and MVPA demonstrated that only girls had a significant and reducing prediction from T1 MVPA to T2 PC. To our knowledge, this is the first study to investigate the dynamic relationships of MSC, PC, and PA among preschoolers over time.

Our findings suggested that although MSC significantly predicted later MSC, there was no evidence of a bidirectional relationship between MSC and PA, which did not support our first hypothesis. The observation in the whole sample regarding T1 MSC predicting T2 MSC was in line with a recent previous study [48]. Indeed, using a sample of 440 elementary school-aged children, Burns et al. [48] reported that children with higher MSC tended to have higher future MSC. However, our finding of no cross-lagged effects between the two variables in the whole sample contradicts previous studies, which demonstrated that greater MSC was positively associated with vigorous-intensity PA in preschool children [9,10,25,48,49,50,51]. The reasons for the inconsistent findings are partially attributable to subject target, varied measurements of PA and MSC across these studies. For instance, Burns et al. [48] aimed elementary school children and utilized pedometers to measure PA and TGMD-3 to measure MSC, while our study used accelerometers and five skills of TGMD-2 (running, hopping, jumping, kicking, and throwing) to investigate preschool children’s dynamic relationships between MSC and PA. TGMD-2 and TGMD-3 are reliable assessments of gross motor skills in children aged 3–10 years, which have shown high reliability test results [38,52]. The TGMD-3 is the updated edition of TGMD-2, which contains three new skills (skipping, underhand throwing, and one-handed striking) and eliminates two skills (leaping and rolling) [52,53] in TGMD-2. While previous study using the sample of preschool children yielded similar evaluation results between TGMD-2 and TGMD-3 [53], future studies are needed to understand the comparisons between the two TGMD editions in diverse contexts. In addition, we observed interesting gender disparities: the negative cross-lagged relationship between MSC and PA was only observed in girls, which is a perplexing finding given the previous study findings [48,54,55]. For instance, Burnes et al. [48] recently reported that MSC is a stronger predictor for later PA among girls than it is among boys. Despite the contradictory findings, our study observed clinically relevant correlation between MSC and MVPA finding in girls. We believe that this finding is still meaningful because girls tend to have lower MSC than boys [22,56] and thus, generally have greater need for MSC development. Yet, considering the gender differences reported in the types of motor skills (locomotor and object control skills) [53,57,58], future research should investigate gender effects in bidirectional relationship between specific MSC and PA.

Furthermore, our total sample data indicated no significant bidirectional or predictive relationship between MSC and PC, indicating that MSC and PC were not considered as significant contributors of each variable. These observations aligned with a recent study that was held in two U.S. elementary schools [43]. However, several previous studies showed inconsistent findings [21,22,59,60]. For instance, Sollerhed et al. [21] reported that children aged 8–12 years with higher PC exhibit better persistence to master a skill, while children with lower PC easily lose interest in the task at hand. Indeed, using a sample of preschool children, Robinson [22] observed a moderated and significant correlation between PC and MSC. Although our findings indicated no evidence of a positive predictor in this relationship in the whole sample, a significant prediction from T1 MSC to T2 PC was observed only in boys. Similarly, LeGear et al. [60] found that preschool-aged boys with greater MSC had higher PC, regardless of types of motor skills. Additionally, Robinson [22] observed a significant correlation between MSC and PC in boys. However, both of these previous studies [22,60] also demonstrated girls’ positive correlation between MSC and PC. It is unclear as to why, unlike these two previous studies, the baseline MSC was predictive of follow-up PC only in boys; however, one of the potential reasons could be the different MSC analysis approach that was adopted in the current study. Le Gear et al. [60] and Robinson [22] used all 12 items (six locomotor skills: run, jump, hop, slide, gallop, and leap; and six object control skills: throw, roll, kick, strike, catch and bounce) of TGMD-2, while the current study selected five gross motor skills (three locomotor skills: run, hop, and jump; and two object control skills: kick and throw). Prior studies [28,29,61] reported that boys had higher motor skill competence, especially object control skills, than girls. Notably, Foulkes et al. [29] showed that preschool-aged boys tend to be more competent at object control skills, such as kicking and throwing. Therefore, a potential reason for the measured MSC gender difference could be attributed to the current study’s MSC measurement, which might have been more favorable to boys than girls.

Lastly, there were no bidirectional relationships observed between PC and PA, and thus, our third hypothesis was not supported. Our findings are consistent with several previous observations in preschool children [23,62], whereas some studies reported a positive association between PC and PA [22,63,64]. A potential reason for the mixed results that were reported in the previous literature could be attributed to young children’s inflated perceived competence. Therefore, when the teacher’s and children’s perceptions of physical competence are compared, both are not always accurate [23,65]. Moreover, in the current study, children’s PA was only measured during the school day; therefore, this current study may not have captured children’s complete daily PA pattern. Since children engaged in structured PA while they were in schools, children’s PA patterns tended to be similar in this study. Hence, the different results compared to some previous studies may be partially attributable to difficulty in accurately assessing children’s perceived competence and the discrepancy in total PA measurement. Although no significant cross-lagged association was observed in the whole sample, the findings from gender-specific analyses indicated that only girls had a negative cross-lagged relationship between T1 PA and T2 PC, which supports the demand to help preschool children to enhance PC for further PA participation given that previous literature [7,15,16,17,18,19,20,21,22,23] suggested the importance of PC in the underlying mechanisms of PA engagement. Yet, few studies support the influence of PA on PC because most of the previous studies focused on investigating the influence of children’s PC on their PA [17,23,64] and the mediating effects of PC on PA [54,66]. Therefore, further research is needed to reveal the cross-lagged relationships between PC and PA. Exploring the bidirectional relationship of PC and PA will be a foundation to understand the underlying mechanisms of PC and PA, which could further the current understanding of the casualty of PC and PA.

Several practical implications can be derived from this study. First, the observations of this study are important given our data provide scientific evidence supporting gender in moderating the relationships between MSC, PC, and PA. Indeed, it was demonstrated that MSC was a significant predictor of MVPA and MVPA was a significant predictor of PC in girls, whereas MSC was reported to be a significant predictor of PC in boys. Therefore, the gender differences that were observed in our study could help with developing effective PA interventions for preschool children so that they can be specifically tailored to gender preferences. It is also worth noting that children of preschool age are an important target for behavioral change strategies, as this age cohort may enhance tracking into the crucial period of adolescence. Hence, our study could contribute to developing effective interventions and strategies that are aimed at encouraging young children to be more physically active.

Our study has several strengths. First, our study used a cross-lagged panel model approach, which allows us to investigate the direction and strength of the associations between PA, MSC, and PC by testing bidirectional relationships between the variables [43]. Second, we used objective measurements to assess PA, MSC, and PC in preschool children. Lastly, a large number of underserved children of minority and low socioeconomic status were targeted in the study. However, several study limitations should be addressed. First, in the current study, ActiGraph accelerometers were only worn during school hours to measure children’s time in MVPA, thus missing children’s actual PA levels outside of school. Indeed, since children performed structured activities during the school day, their PA was identical for the most part, which could partially explain why the proposed relationships of PA to other health-related outcomes were not observed. Therefore, future research should use the same objective measurement tools to assess preschoolers’ daily PA levels, as well as light physical activity levels or sedentary behaviors to provide further insights. Additionally, the generalizability of the study findings may be limited due to the small sample size and uneven distribution of racial backgrounds, and the relatively short length of the study observation. Hence, longer trials with a large sample that includes a diverse population are recommended for future studies. Lastly, the current study examined MSC using the total average score of five skills (the running, hopping, and jumping locomotion skills and the kicking and throwing objective control skills), thus missing the specific gender effects in different types of motor skills. Hence, future research should investigate bidirectional relationships between types of motor skills and multiple PA-related psychological factors to discern which types of motor skills need to be improved in preschool-aged boys and girls.

## 5. Conclusions

In conclusion, preschool children’s MSC significantly predicted future MSC over 8 weeks and gender differences were demonstrated in all three cross-lagged panel models. Despite the non-significant bidirectional relationships, such findings provided initial evidence for reciprocal, mutually beneficial relationships between PA and its important correlates (i.e., MSC and PC) in preschool children. Considering the gender differences in the predictive relationships between MSC, PC, and PA and targeting underserved young children have important practical implications for PA researchers who are aiming to develop and test health-behavior interventions that can lead to reduce children’s low PA behaviors and health disparities in schools. However, future studies employing larger trials with a greater number of participants and considering the types of motor skills (i.e., locomotion skills and objective control skills) are warranted.

## Figures and Tables

**Table 1:** Descriptive statistics (means and standard deviations).

	Total Sample (*n* = 61)	Girls (*n* = 31)	Boys (*n* = 30)
Age (years)	4.44 (0.46)	4.51(0.47)	4.38 (0.44)
BMI (T1)	15.91 (1.61)	15.55 (1.51)	16.29 (1.64)
BMI (T2)	15.72 (1.67)	15.23 (1.42)	16.23 ** (1.77)
BMI z-score (T1)	−0.20 (1.28)	−0.00 (1.23)	0.41 (1.31)
BMI z-score (T2)	0.04 (1.41)	−0.25 (1.38)	0.34 (1.42)
Total MVPA (T1)	38.96 (9.71)	36.77 (9.26)	41.22 (9.78)
Total MVPA (T2)	41.03 (6.30)	38.68 (6.43)	43.47 *** (5.23)
Perceived competence (T1)	3.21 (0.45)	3.21 (0.49)	3.2 (0.423)
Perceived competence (T2)	3.16 (0.53)	3.09 (0.52)	3.24 (0.55)
Motor skill competence (T1)	32.26 (4.98)	31.26 (5.13)	33.3 (4.68)
Motor skill competence (T2)	35.03 *** (4.56)	35 (4.14)	35.07 (5.04)

Note: BMI, body mass index; MVPA, moderate-to-vigorous physical activity; T1 stands for the baseline time, i.e., before the 8-week study period; T2 stands for the follow-up time, i.e., after the 8-week study period. ** and *** denote a statistical difference between the time points, where ** *p* < 0.05 and *** *p* < 0.01.

## Data Availability

The data that are presented in this study are available on request from the corresponding author. The data are not publicly available due to the lack of an online server for data storage.
